# Genetic diversity, functional properties and expression analysis of *NnSBE* genes involved in starch synthesis of lotus (*Nelumbo nucifera* Gaertn.)

**DOI:** 10.7717/peerj.7750

**Published:** 2019-09-25

**Authors:** Fenglin Zhu, Han Sun, Ying Diao, Xingwen Zheng, Keqiang Xie, Zhongli Hu

**Affiliations:** 1 College of Life Sciences, Wuhan University, Wuhan, China; 2 State Key Laboratory of Hybrid Rice, Wuhan, China; 3 Hubei Lotus Engineering Center, Wuhan, China; 4 Guangchang Bailian Institute of Jiangxi Province, Guangchang, China

**Keywords:** Amylopectin, Affinity activity, Lotus, NnSBE, Relative expression, Homozygous haplotype

## Abstract

**Background:**

Starch branching enzyme (SBE) is one of the key enzymes in starch biosynthetic metabolism, determining amylopectin structure.

**Methods:**

Full length coding sequences (CDS) of * SBE* genes were cloned using reverse transcription PCR (RT-PCR) technology, and neighbor-joining (NJ) tree was used for phylogenetic analysis. Single nucleotide polymorphisms (SNPs) were determined to assess the genetic polymorphisms and variation indexes between individuals and clusters. Quantitative real time PCR (qRT-PCR) was performed to analyze the spatial and temporal expression of *NnSBE* genes. The effect of *NnSBE* genes on amylopectin’s fine structures was explored using affinity and the enzyme activity analysis of two isoforms in amylopectin and amylose.

**Results:**

In this study, two SBE family genes, *NnSBEI* and *NnSBEIII*, were identified in lotus (*Nelumbo nucifera* Gaertn.). Phylogenetic analysis sorted NnSBEI into SBE family B and NnSBEIII into SBE family A. UPGMA phylogenetic tree divided 45 individuals of lotus into three classes. The homozygous haplotype (A G G A G) of* NnSBEIII* was observed in seed lotus. During the seed embryo development stage, *NnSBEIII* reached the peak in the middle of the development stage, while *NnSBEI* increased in the mid-late developmental stage. The different affinity activity of the two isozymes binding amylopectin and amylose assay indicated NnSBEI has higher activity and wider affinity.

**Discussion:**

Genetic diversity showed that *NnSBE* genes received artificial selection during the process of cultivation and domestication in lotus seeds. Furthermore, the expression pattern and affinity activity analysis indicated that *NnSBE* genes were related to the chain length of amylopectin.

## Introduction

Lotus (*Nelumbo nucifera* Gaertn) is an ancient perennial aquatic plant and important crop in Asia. Archaeological research has estimated that the history of lotus is more than 7,000 years old, and it has been cultivated for more than 2000 years (Guo, 2009; Shen-Miller, 2002). In general, lotus are grouped into three clusters corresponding with the three different important organs, namely ornament lotus, seed lotus and rhizome lotus, respectively (Guo, 2009; Wu et al., 2007). As a type of aquatic plant with high photosynthetic efficiency and high carbon conversion, lotus has high starch content. Because of their high amount of starch, the edible rhizomes and seeds play a key role in a daily diet and cultural activities (Chen et al., 2007). Studies have shown that fresh rhizomes, on average, are comprised of 10–20% starch in their total fresh weight, while the amount is 40–60% in mature seeds (Shen-Miller et al., 1995).

Starch is an important polysaccharide and the major form of carbohydrate storage in plants (Slattery, Kavakli & Okita, 2000). It is a necessary part of the human diet in terms of nutrition and calories. Starch is comprised of two glucan polymers, amylose and amylopectin, which have different characteristics because of their starch molecular structure. Amylose and amylopectin synthesis are regulated by the coordinated action of a series of enzymes. AGPase produces substrate that plays a role in the synthesis of amylose and amylopectin. Amylose synthesis is mainly controlled by granule-bound starch synthase (GBSS), while amylopectin is generated by the successive work of starch synthase (SS), starch branching enzyme (SBE), and debranching enzyme (DBE) (Fujita et al., 2008; Jeon et al., 2010; Subasinghe et al., 2014; Tetlow Ian & Emes Michael, 2014).

Starch-branching enzymes, formerly known as Q-enzymes, have dual catalytic functions and determine the structure of amylopectin. SBE cleaves the internal a-1,4 linkage from polyglucans and then transfers the reducing ends to C-6 hydroxyls to generate a-1,6-branch linkage in the liner chain (Martin & Smith, 1995). Their catalytic function not only catalyzes the formation of a new branch, but they also add new nonreducing ends in the starch molecule. Thus, SBE determines the branching pattern in amylopectin, which is believed to affect the fine structure of plants and influence the amount of starch (Satoh et al., 2003).

SBEs have been researched in various plants. In particular, two cDNA coding *PsSBE*s were identified from the embryo of *Pisum sativum* by Burton Rachel et al. (1995). Later, two or three SBE members were identified in various plants and were classified into SBE A and SBE B based on phylogenetic analysis. SBE A and SBE B play different roles in the influence of amylopectin synthesis. In the process of starch synthesis, the SBE A family tends to amylopectin, while SBE B family show higher affinity for amylose. SBE A prefers amylose as a substrate and predominantly transfers relatively longer chains (>14DP), while SBE B tends to amylopectin and transfers shorter chains (<14DP) (Guan et al., 1997). In addition, different gene expression patterns of *SBE A* and *SBE B* were detected with a range of species, indicating that *SBE B* is expressed earlier than *SBE A* in the development stage (Gao et al., 1996; Larsson et al., 1998; Morell et al., 1997; Mutisya et al., 2003).

To fit the rapid development of the lotus processing industry, the work of breeding lotus with high starch content is extremely urgent. Therefore, many studies have focused on the starch of lotus. The *ADPase* and *GBSS* genes of lotus were isolated and characterized, but little information is currently available about the *NnSBE* gene (Cheng et al., 2014; Liang-Jun et al., 2006; Lu et al., 2012). For researching the *NnSBE*s further, this study undertook the isolation of cDNA and genomic clones by encoding two related *SBE* genes from lotus. Then, genomic variation and evolution were analyzed using DNA and protein sequences of the two NnSBEs. Expression patterns and the affinity of two isozymes were described, as well as the enzyme activity of both NnSBEs in development and different tissues. These data provide valuable information for understanding the processes involved in starch synthesis, and offer some fundamental information for further study about improving the edible quality of lotus.

## Material and Methods

### Plant material and treatments

*Nelumbo nucifera* cv. Taikong lotus 36, the highest strain of selective lotus breed after space mutagenesis, was selected in this study (Wu et al., 2007). Seeds were sprouted by soaking in water for germination. Five days after, plants were provided with 50 cm depth pots in a greenhouse for the entire growing season. Functional leaves, petiole, rhizomes and roots were separately collected in the 8th week, 10th week, 12th week. Seeds from plants at 12, 16, 20, 24 and 28 DAF (days after fertilization) were also collected from the Taikong lotus 36 in the genetic experimental base of Wuhan University. Various materials from lotus were quick-frozen in liquid nitrogen, then stored at −80 °C for next manipulation. The fresh leaves of 45 lotus individuals (Supplement 1) were collected from the genetic experimental base of Wuhan University and stored in silica gel (Sinopharm, China).

### RNA isolation and cDNA synthesis

Total RNA and genomic DNA were isolated from plant tissue samples using a plant RNA extraction kit and Plant Genomic DNA Kit (TIANGEN, Beijing, China), according to their respective operation manuals. The quantities were subjected to 1% agarose gel electrophoresis and the results were analyzed using a UV Transilluminator (Eppendorf, Hamburg, Germany). The first-strand cDNA of all materials was constructed using the FastKing RT Kit (with gDNase) (TIANGEN). The products were stored at −20 °C for later use.

### Characterization of a genetic polymorphism

Genetic polymorphism of *NnSBE* cDNA from 45 individuals of lotus were also analyzed using the gene-specific primers. PCR reactions were conducted in 50 µl volumes containing two µl of total cDNA, five µl of 10 ×PCR buffer, five µl of two mM dNTPs mixture, three µl of 25 mM MgSO_4_, 1.6 µl of 10 pM of each primer, one µl of one U/µl KOD DNA polymerase (TOYOBO, Osaka, Japan) and 30.8 µl ddH_2_O, for a total volume of 50 µl. Amplification conditions followed the two-step amplification procedure: 94 °C for 2 min, 36 cycles of 98 °C for 10 s, (Tm)  °C for 30 s, and 68 °C for 1 min. The sizes of the PCR products were assessed using 1.0% agarose gel electrophoresis. PCR products were sequenced by Sanger sequencing (Augct, Beijing, China). Sequences were aligned by DNAman to find molecular markers.

### Bioinformatics analysis

All sequence data were obtained from the National Center for Biotechnology Information (NCBI) GenBank database (http://www.ncbi.nlm.nih.gov/). All primers were designed by Primer Premier 5.0v. Genomic structures were performed with the program Gene Structure Display Server 2.0 (http://gsds.cbi.pku.edu.cn/) (Hu et al., 2015). The GO (Gene Ontology, http://www.geneontology.org) was used for annotation of gene products. The conserved domains were predicted by Pfam server (http://pfam.xfam.org/search/sequence;) (Finn et al., 2016). All sequences were aligned using Cluxal-X(Thompson et al., 1997) and DNAman. Subsequently the phylogenetic neighbor-joining tree was generated by MEGA6.0v (Tamura et al., 2013). The bootstrap consensus tree inferred from 1,000 replicates was taken to depict the evolutionary history of the analyzed taxa. The *H*_O_, *H*_E_, Shannon Index was calculated by popGen32 (Nei, 1978; Nei & Li, 1973; Nei & Li, 1979). A dendrogram of the cluster analysis was based on Nei’s genetic distance using the UPGMA method.

### Real-time PCR analysis

The cDNA of reverse transcription was diluted for 1:10 and used for RT-PCR and qRT-PCR assays. A house-keeping gene, *CYP* (cyclophilin, GenBank accession no. EU131153), was selected as the reference gene in this experiment. The primers of *CYP* were based on the sequence of CDS and designed by Primer Primer 5.0 (Table 1). The total PCR reaction mixture contained 2 µl cDNA (1:10 diluted), 0.4 µl forward primer (10 M), 0.4 µl reverse primer (10 M), 10 µl 2 ×SYBR qPCR Master Mix (with ROX Premixed) (Vazyme, China) and 7.2 µl ddH_2_O, for a total volume of 20 µl. For the qRT-PCR experiment, the two-step amplification procedure was used: 95 °C for 10 min, followed by 40 cycles of 95 °C for 15 s and 60 °C for 1 min. The relative gene expression data was calculated using the 2^−ΔΔCt^ method with the guidance of the StepOne software v2.1 (ABI, US). The experimental design followed MIQE (minimum information for publication of quantitative real-time PCR experiments) (Bustin et al., 2010; Bustin et al., 2009). All measurements were processed three times for biological and technical paralleled repetition.

**Table 1:** Primers used in qRT-PCR.

Gene	Sequence forward & reverse primers (5′-3′)	Amplicon length (bp)	*R*^2^	Primer efficiency
q*CYP*-F	GTACCCAGAAGAATGCCCTA	102	0.998	96.222
q*CYP*-R	ATGAAGCCCTTGATGACTCG			
q*NnSBEI*-F	GTAGACCATTTCACATCGC	114	0.999	93.132
q*NnSBEI*-R	TAATAAGCCACACATGTACGAG			
q*NnSBEIII*-F	TATGCATGGCTAGTTCCAC	110	0.999	86.246
q*NnSBEIII*-R	TTATGCCAAAATGCCTCGT			

### Construction of plasmids

The full length CDS of *NnSBEI* and *NnSBEIII*, which were cloned from lotus, were subcloned into the plasmids pET-28a and pET-32a respectively. The pET-28a-*NnSBEI* was generated using the following primers: 5′-CCCAAGCTTATGTACAGTTTTTCTGGGT-3′(HindIII site underlined), and 5′-CATGCCATGG TCAGTCATCCAATCCCA-3′(Nco I site underlined). The pET-32a-*NnSBEIII* was obtained by primers: 5′-CGGGATCCATGG CTACTACAGTTGCGCT-3′(BamHI site underlined) and 5′-GCGATATCTCATATTCG CAAAATCCGAG-3′(EcoR V site underlined). The full length and sequence of the nucleotide acid of the inserted gene in the extracted recombinant plasmid were completely identical to the genes. Then, the different recombinant plasmids were transformed into Escherichia coli BL21 (DE3) (TransGen Biotech, Beijing, China) for expressing and into DH5α for storing.

### Enzyme activity assay

Cheng et al.’s protocol of measuring SBE activity was used in this study (Cheng et al., 2001). First, total enzyme isolation: about 0.4 g powder of plant material was added into a 4 ml HEPES–NaOH (PH7.5) buffer. After proper shaking, the tube was centrifuged at 10,000 g for 15 min, and supernatant was extracted to check the enzyme activity of SBE. We took 100 µl dilution, added 1,280 µl of HEPES–NaOH (PH7.5) buffer, and 120 µl 0.75% of soluble starch. Then we incubated the total reaction mixture at 37 °C, and gave it a water bath for 20 min. Next, we gave it a boiling water bath for 1 min to stop the reaction, diluted the mixture with two ml water (containing 0.2% HCl), added 150 µl of an iodine solution (0.1%I_2_-1%KI), blending it at room temperature for 10 min. The boiled crude enzyme was used as the control. Finally, we measured absorbance at 660 nm. The enzyme activity of SBE was expressed as a percentage of decrease in absorbance at the wavelength of 660 nm to be compared with the control. All measurements were processed three times for biological and technical paralleled repetition.

### The affinity of two isozymes that bind amylopectin and amylose assay

Recombinant proteins were expressed in the Escherichia coli BL21(DE3) cells. The transformed cells were grown in LB at 37 °C until the OD600 reached 0.6–1.0, using IPTG to induce expression with a final concentration of 0.5 mM, and were further incubated at 16 °C for 12 h. Cells were harvested by centrifugation at 12,000 rpm for 5 min at 4 °C. The products were suspended in a buffer containing 10 mM PBS in the radio of 200:1 (ml/g), and cells were disrupted (Diagenode, Liege, Belgium) by sonication for 25 min and centrifuged for 5 min at 4 °C. We took 30 µl supernatant, added 0, 12.5, 16, 20, 25, 35 µll 0.75% amylopectin (Sigma-Aldrich, St. Louis, MO, USA) or amylose (Sigma), and added HEPES–NaOH (PH7.5) buffer into 90 ml. Then the total reaction mixtures were incubated at 37 °C for 30 min. We diluted the mixture with 100 µll water (containing 0.2% HCl), and added 10 µll iodine solution (0.1%I_2_-1%KI), blending at room temperature for 10 min. Finally, we measured absorbance of amylopectin at 560 nm and amylose at 620 nm. The blank pET-28a and pET-32a which were transformed into BL21 and cultured in the same condition were used as the control. The affinity of the two isozymes that bind amylopectin and amylose were measured by enzyme activity. All measurements were processed three times for technical repetition and 2∼3 paralleled repetition.

## Results

### Cloning of *NnSBE* genes

**Figure 1:**
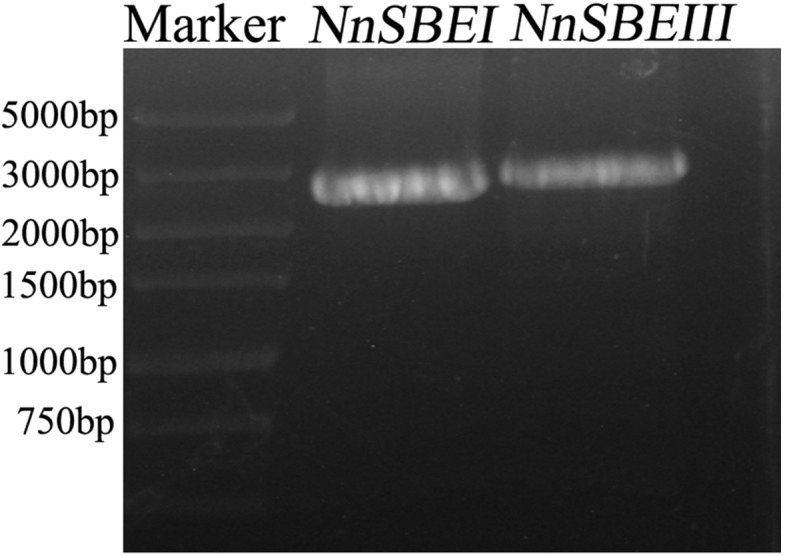
The amplication of cDNA fragments of *NnSBE*s. The amplication of cDNA fragments of *NnSBE*s *NnSBEI* and *NnSBEIII* using RT-PCR experiments. The products were analyzed by electrophoresis on 1.0% agarose gels, DL 5000 DNA marker.

**Figure 2:**
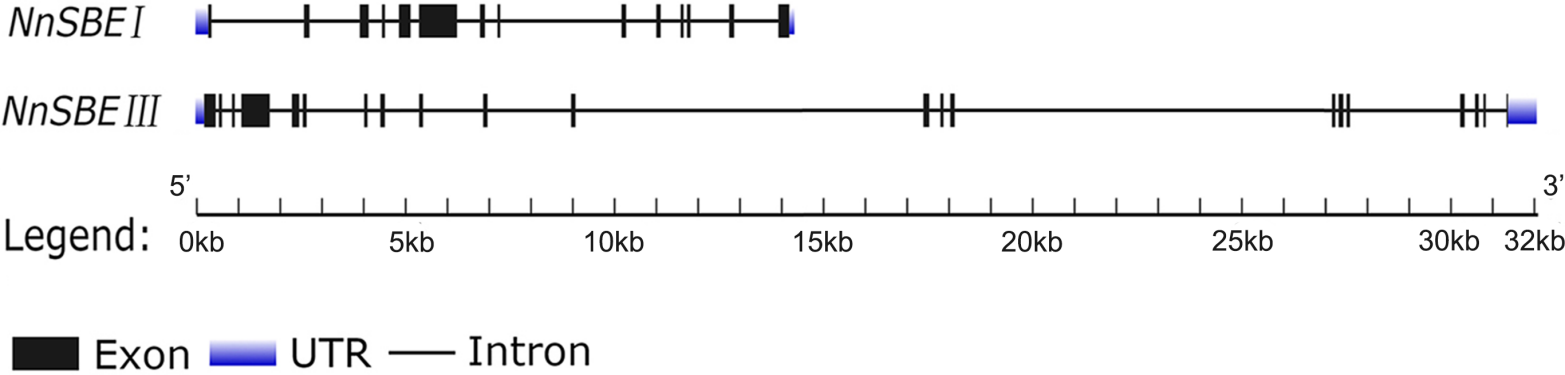
The gene structure of *NnSBEI* and *NnSBEIII*. The blue boxes represent UTR, black boxes represent the exons, thick line represent intros.

Based on the reference sequence of whole-genome sequencing, the CDS of two *SBE* genes were obtained using RT-PCR technique from the embryo of the lotus seed. The CDS of *NnSBEI* (FJ592190.1) was 2,577 bp, and the CDS of *NnSBEIII* (XM_010254179) was 2691bp (Fig. 1). Complete genomic structures of *NnSBEI* (comprising of 14 exons and 13 introns) and *NnSBEIII* (comprising of 21 exons and 20 introns) were separately distributed over 14.3 and 32.1 kb. The gene structures are shown in Fig. 2 and the gene information is shown in Table 2. The cDNA of *NnSBEI* showed the highest (85%) identity with *Castanea mollissima*, and 80–84% identify with *SBEI* genes of other plant species, while *NnSBEIII* showed the highest (85%) identity with *Juglans regia* and 80–84% identity with the others.

**Table 2:** Gene information of *NnSBEI* and *NnSBEIII*.

Gene name	Gene ID	Genomic length(kb)	ORF length (bp)	Number of exons	Number of intros	Number of amino acid (Aa)
*NnSBEI*	104,603,742	14.3	2,352	14	13	783
*NnSBEIII*	104,594,060	32.1	2,691	21	20	896

The deduced protein of NnSBEI comprised 858 amino acids with a predicted molecular mass of 97.144 kDa, and NnSBEIII comprised 896 amino acids with a predicted molecular mass of 103.135 kDa. GO analysis indicated NnSBE participated in biological processes (GO:0005978) and one molecular function (GO:0043169; GO:0003844; GO:0004553). Pfam analysis showed that NnSBEs have three domains of secondary structure: the central catalytic A domain, an N-terminal domain and the C-terminal domain. The central catalytic A-domain of SBEs is *α*-amylase which covered four conserved amino acid regions and six active sites spread in those conserved regions respectively. The characterization of catalytic A-domain showed significant homology (47.83%) between NnSBEI and NnSBEIII, but highly dissimilar sequences in N-terminus (21.84%) and C-terminus (26.47%).

### Phylogenetic analysis

**Figure 3:**
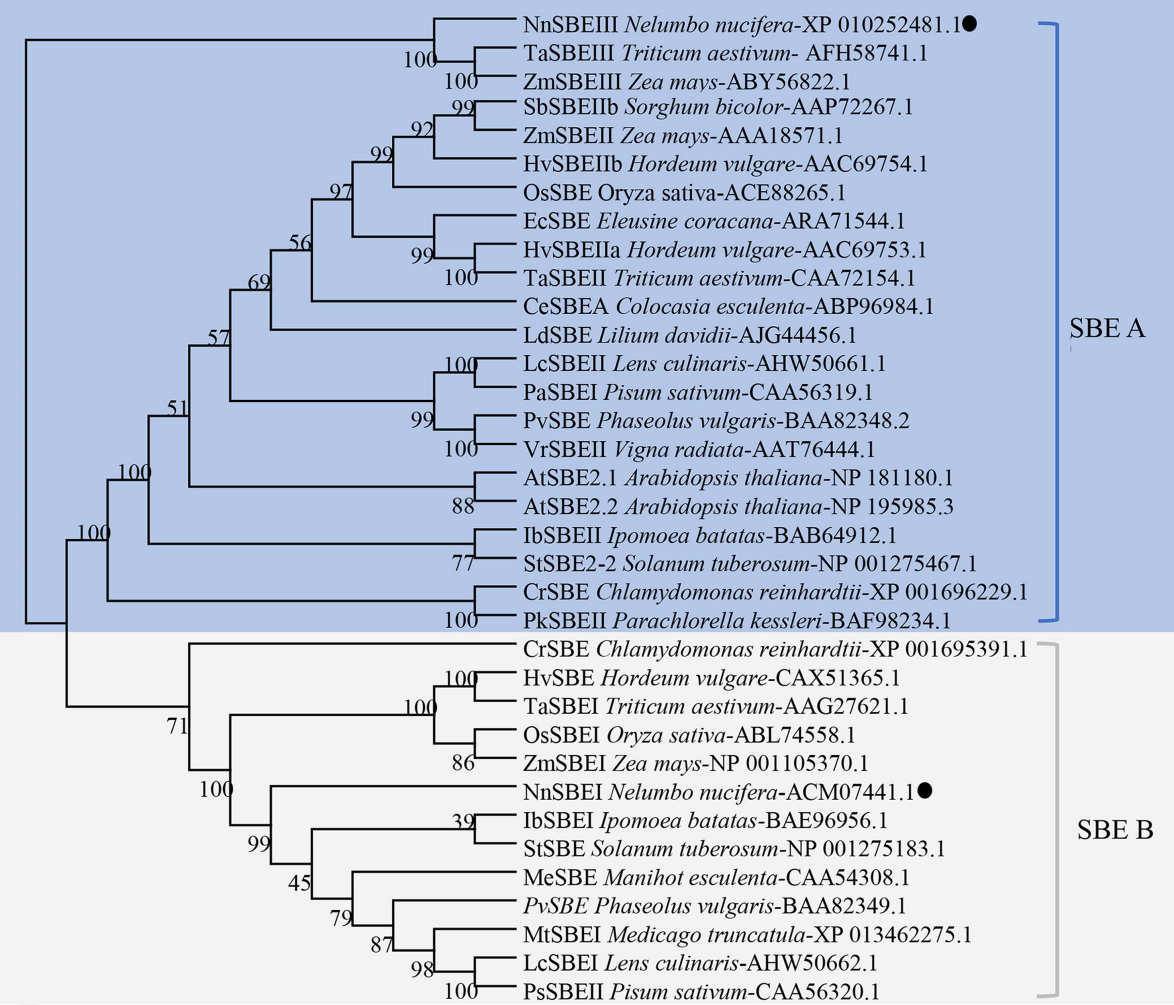
Phylogenetic analysis of the SBEs family. The dendrogram was constructed using MEGA6.0 software with the neighbor-joining method. Sequences aligned included the SBE protein sequences of 20 other species, which identified or predicted from NCBI database were used for phylogenetic analysis. Different color regions represent two subclades of SBEs: the blue region cover SBE A family, and the gray cover SBE B family. The NnSBEs were labeled with “●”.

SBE protein sequences from 20 other species were used for phylogenetic analysis, shown in Fig. 3. The NJ phylogenetic tree indicates that SBEs in these plants could be classified into two families: SBE A and SBE B. SBE A was further divided into four classes: algae SBEII, dicot SBEII, monocot SBEII and SBEIII, and SBE B was subdivided into algae, dicot SBEI, monocot SBEI. NnSBEI belonged to the dicot SBEI of family B; NnSBEIII belonged to the SBEIII class of family A. Evolutionary divergence reveal that SBEIII came before SBEI, and differentiation of NnSBE occurred before that of the other dicots, but later than the monocots.

### Characterization of genetic diversity

**Figure 4:**
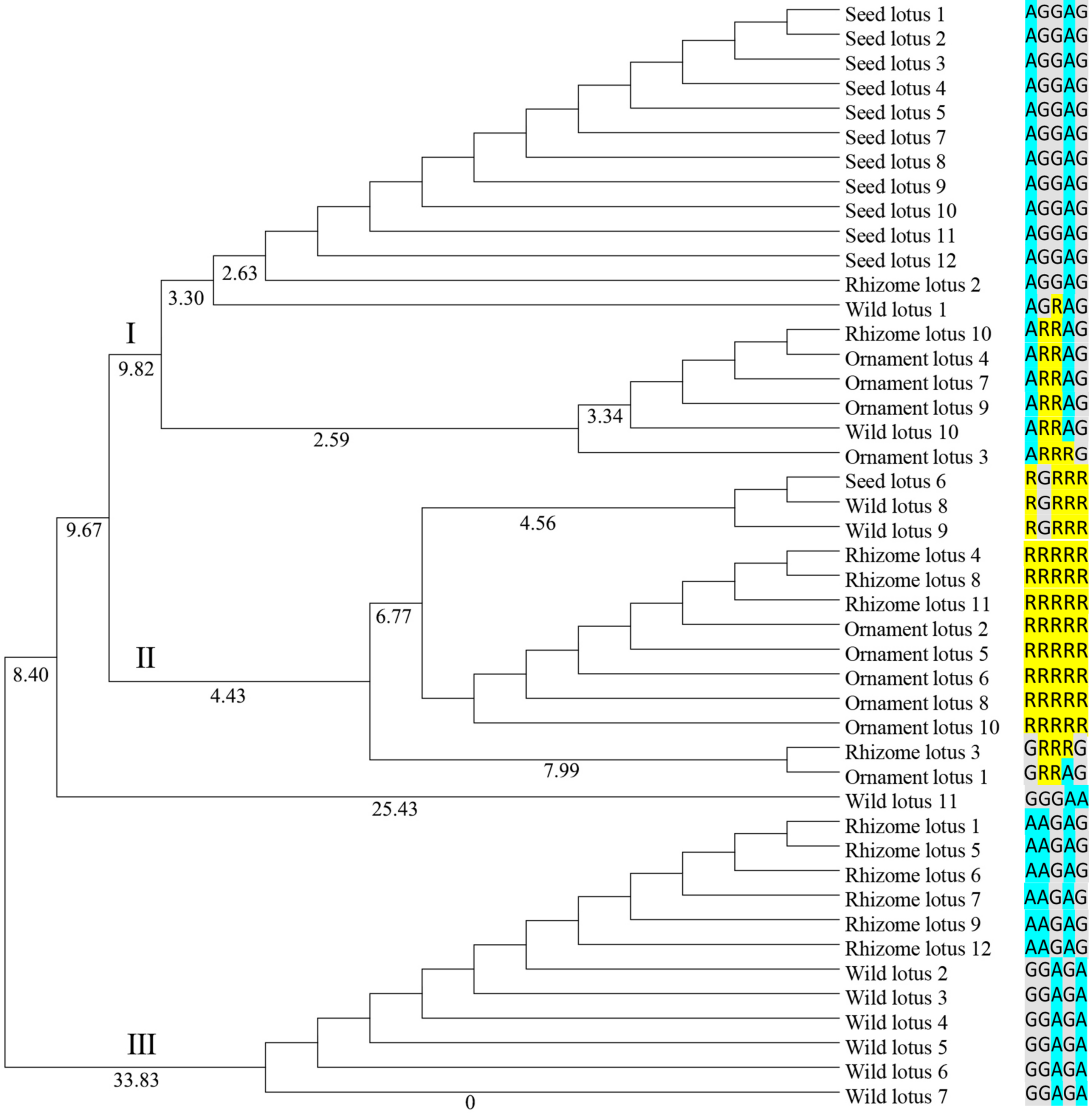
Dendrogram of 45 individuals lotus based on SNP data of *NnSBEIII*. Dendrogram based Nei’s genetic distance and the UPGMA method was using for Cluster analysis. The 45 individuals of lotus were copolymerized into three classes. The number of individuals were shown in the Supplemental Information 1 and SNPs data were shown as colormetric distinction.

Genetic diversity was identified based on the ORF of two *NnSBE* genes from 45 lotus individuals belonging to four clusters (Supplement 1). This revealed six polymorphic sites from the coding region: five SNPs in *NnSBEIII* and one in *NnSBEI*. The SNPs of *NnSBEIII* was analyzed and the detailed parameters of genetic diversity are listed in Table 3. Three SNPs resulted in missense mutations which changed the amino acid sequence, while the other two SNPs were synonymously mutated. The observed heterozygosity ranged from 0.2 to 0.4444, and the expected heterozygosity ranged from 0.4012 to 0.4583. The Shannon-Wiener Index ranged from 0.5908 to 0.6508. In further association analysis of starch content (amylopectin, amylose, total starch) in lotus seeds, no SNP showed highly significant associations. Total of 12 genotypes were detected in those 45 individuals, each cluster had different several kinds of genotypes (Supplemental Information 1). A homozygous genotype (AA GG GG AA GG) of *NnSBEIII* was observed in most individuals in seed lotusand a haplotype (A G G A G) was identified from seed lotus. Cluster analysis according to the UPGMA method is shown in Fig. 4. The 45 individuals of lotus were copolymerized into three classes. The first class can be divided into two subclasses with a similarity coefficient of 9.8, and most seed lotus showed together in a same subgroup. These results paved a way to apply the useful allelic variations or gene haplotypes in cultivar lotus and quality breed programs.

**Table 3:** Genetic diversity based on SNPs of *NnSBEIII* in the test population.

SNPs of *NnSBEIII*	Aa	Allelic frequency		Genotypic frequency		*H*_O_	*H*_E_	Shannon index
c.282 G>A	M	A	0.6778	AG	0.2444	0.2444	0.4368	0.6285
		G	0.3222	AA	0.5556			
				GG	0.2			
c.535 G>A	E/K	A	0.3111	AG	0.3556	0.3556	0.4286	0.6200
		G	0.6889	AA	0.1333			
				GG	0.5111			
c.880 G>A	V/L	A	0.3556	AG	0.4444	0.4444	0.4583	0.6508
		G	0.6444	AA	0.1333			
				GG	0.4222			
c.2308 G>A	O/N	A	0.7000	AG	0.2889	0.2889	0.4200	0.6109
		G	0.3000	AA	0.5556			
				GG	0.1555			
c.2559 G>A	G	A	0.2778	AG	0.2000	0.2000	0.4012	0.5908
		G	0.7222	AA	0.1778			
				GG	0.6222			

**Notes.**

H_O_ the observed heterozygosity *H*_E_ the expected heterozygosity

### Expression pattern

**Figure 5:**
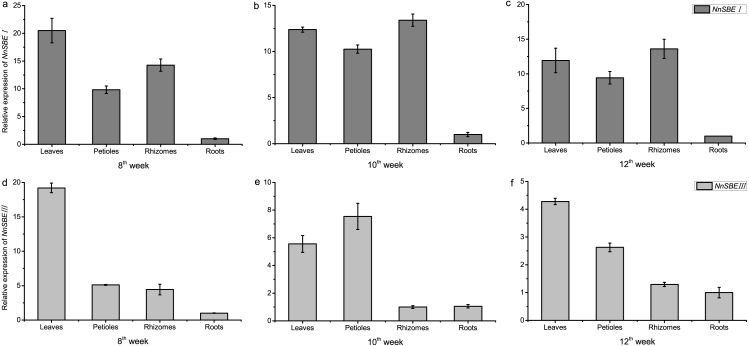
Tissue-differential expression of the *NnSBE*s. The column chart confirmed expression pattern of leaves, petiole, rhizomes, roots which collected in 8th week after sowing at the early swelling stage (A, D), collected in 10th week after sowing at the middle swelling stage (B, E), collected in 12th week after sowing at the later swelling stage (C, F) in lotus. The gray boxes represent *NnSBEI* and the lightgray represent *NnSBEIII*. Error bars indicate standard error (*n* = 3) (*p* < 0.05).

**Figure 6:**
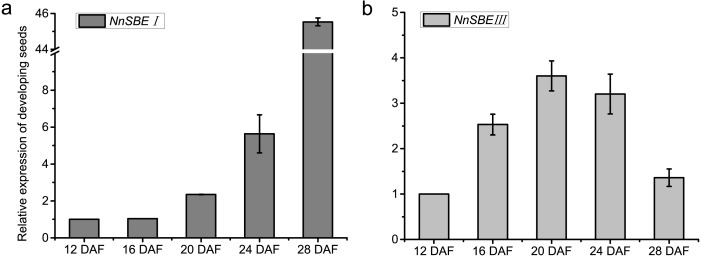
Expressed tendency of the *NnSBE*s during developing seed. Relative expression of the *NnSBE*s were analyzed at 12, 16, 20, 24, 28 DAF. The gray boxes represent *NnSBEI* (A) and the light gray boxes represent *NnSBEIII*. (B) Error bars indicate standard error (*n* = 3) (*p* < 0.05).

The temporal and spatial expression of both genes were analyzed to investigate their expression patterns. The results of qRT-PCR demonstrated that the transcriptional expression level of *NnSBEI* and *NnSBEIII* was detectable in all tissues. Tissue-specific expression analysis showed that the highest transcript levels of *NnSBEI* and *NnSBEIII* were observed in leaves. The transcript level of *NnSBEI* was higher in rhizomes while *NnSBEIII* expressed strongly in petioles. The expression profile of *NnSBEI* in the rhizome showed strong temporal differences, and the relative expression level increased gradually from initial development to late stage. *NnSBEIII* showed temporal differences in the petiole, which enhanced in the middle and decreased in the late stage (Fig. 5). During the seed embryo developing stage, *NnSBEI* and *NnSBEIII* expressed significant differences. Transcripts of *NnSBEIII* reached the peak at 20 DAF in the middle of development stage, and then decreased gradually, while *NnSBEI* increased in 16 DAF to 28 DAF and expressed strongly in the mid-late developmental stage (Fig. 6). In the peak stage, *NnSBEI* expressed ten times higher than *NnSBEIII*.

### Dynamic changes of enzyme activities

**Figure 7:**
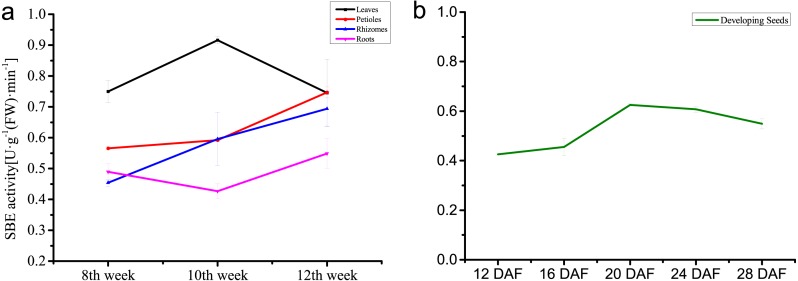
Enzyme activity of NnSBE. Tissue-differential enzyme activity of NnSBE in leaves, petiole, rhizomes, roots which collected in 8th week after sowing at the early swelling stage, 10th week after sowing at the middle swelling stage, 12th week after sowing at the later swelling stage, and the different colors (black, red, blue, purple) represent various organs(leaf, petiole, rhizome and root) in an orderly way from top to bottom (A). Enzyme activity of developing seed of NnSBE at 12, 16, 20, 24, 28 DAF, the green lines represent seed (B). Error bars indicate standard error (*n* = 3) (*p* < 0.05).

The dynamic changes of enzyme activity of the SBEs were analyzed in different temperatures, tissues and developing stages. Incubation in different temperatures revealed that the highest enzyme activity was generated under 37 °C. The enzyme activity of inter-organizational measurement and assessment showed the highest catalytic activity in leaves, followed by rhizomes and petioles which increased steadily, and activity in the roots was the weakest. During seed development, enzyme activity increased rapidly, and the activity peak appeared at 20 DAF, then decreased slightly (Fig. 7).

### The affinity of two isozymes that bind amylopectin and amylose

**Figure 8:**
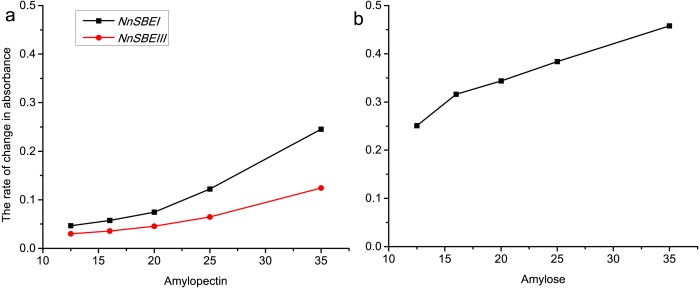
The rate of change in absorbance about NnSBEI and NnSBEIII reacted among different substrate concentration. The substrate is amylopectin (A); the substrate is amylose (B). The black lines represent NnSBEI; the red line represents NnSBEIII.

To explore the affinity activity, recombinant DNA techniques were used to generate the pET-28a-*NnSBEI* and pET-32a-*NnSBEIII* plasmids. Testing of the inducible expression vector showed that both proteins were soluble, and the apparent molecular weights of pET-28a-NnSBEI and pET-32a-NnSBEIII were about 105 kDa and 120 kDa (with His tag) respectively. The affinity activity of NnSBEI and NnSBEIII to amylopectin and amylose were assayed at various starch concentrations. As we can see in Fig. 8, the activity of both isozymes correlated with the types and concentration of substrate. Rate of reaction changed with the increase of the substrate concentration until the enzyme was saturated. NnSBEI showed higher affinity activity when the substrates were amylose and amylopectin. However, another isozyme, NnSBEIII, only worked on amylopectin, and the branching efficiency was only half of NnSBEI.

## Discussion

Starch is an important edible component in lotus, and the starch branch enzyme is involved in the synthesis of amylopectin. In this study, we focused on the SBE family genes in lotus, and revealed two isoforms of *NnSBE*s: *NnSBEI* and *NnSBEIII*. In this study, SBE phylogenetic tree included the SBE gene families from algae, monocots and dicots according to the protein sequences of the sequenced species. Two or three SBE isoforms were isolated from different plants, and divided into two families: family A and family B (Fig. 4). NnSBEI belonged to family B, which has been reported in a range of species. NnSBEIII belonged to family A, which has only been identified in maize, wheat, rice and lotus, and little research has been done about this isoform (Kang et al., 2013; Tian et al., 2009).

Over the last decades, many investigations have been devoted to exploring genomic variation and evolution among different germplasms. Whole genome re-sequencing reveals the evolutionary patterns of sacred lotus, rhizome lotus had the lowest genomic diversity and a closer relationship to wild lotus, whereas the genomes of seed lotus and ornament lotus were admixed (Huang et al., 2018). In this study, genetic diversity and genetic variation of *NnSBEIII* were investigated among 45 individuals from four lotus subgroups. Wild lotus had the higher genomic diversity, rhizome lotus was admixed with wild and flower lotus respectively, and lotus seed formed a homozygous single haplotype, which might be the result of continuous selection in the cultivation process of lotus in the long-term evolution process (Doebley, Gaut & Smith, 2006; Yue, Melamud & Moult, 2006). This different performance induces speculation that the *NnSBE* gene has more effect on starch accumulation in seed lotus, but less in rhizome lotus.

Starch synthesis and accumulation are closely related to photosynthesis. It was found that the *AGPase* and *GBSS* genes of lotus, related to starch synthesis, were expressed higher in leaves (Cheng et al., 2014; Lu et al., 2012). As the tissue of photosynthesis, leaves support the first step of synthesizing sugar and meet the carbon demand faster and it is the major tissue for accumulating transient starch. The results of qRT-PCR showed that the two *NnSBE*s were expressed throughout plant tissues. *NnSBEI* was strongly activated in the leaves and petiole; *NnSBEIII* was highly expressed in the leaves and stems (Fig. 4). The transcriptional level kept rising during the swelling stage in the petiole and rhizome, which represent the photosynthetic tissue and storage tissues respectively. It is possible to adapt to the synthesis of transient starch in photosynthetic tissue, and it is consistent with reserve starch synthesis in storage organs during starch rapid development. This spatial and temporal variation from top to bottom may be related to the process and the transport of starch synthesis to achieve higher efficiency of starch accumulation. Such expression patterns of temporal and spatial controls have also been found in many other species (Tetlow, 2010). This spatial and temporal expression pattern of *NnSBE*s help amylopectin to adapt requirements at different developmental stages.

During evolution, SBE A family is inclined to the amylopectin branch, while SBE B family is more sensitive to amylose. Likewise, this experiment showed that NnSBEI has higher catalytic activity for amylose and amylopectin, while NnSBEIII expressed catalytic activity only when the substrate was amylopectin. Protein sequences of the SBE genes among a range of species revealed that the SBEI and SBEIII subunits showed homology in their *α*-amylase catalytic domain, but highly dissimilar sequences in N-terminus and C-terminus. Construction of chimeric enzymes out of maize branching enzymes found that the N-terminal determined the specificity of the transferred chain length, and the C-terminal domain participates in the specificity of the substrate (Commuri & Keeling, 2001; Hong, Mikkelsen & Preiss, 2001; Kuriki, Stewart & Preiss, 1997). Therefore the N-terminal region of SBE is essential for maximum enzyme activity and thermostability (Hamada et al., 2007), and C-terminus determines both substrate preference and maximal catalytic activity (Hong & Preiss, 2000). We speculated that the difference between enzymatic characteristics of the two subunits are determined by the N-terminus and C-terminus. These differences met the requirements of amylopectin to produce different grades of branches and different sizes of side chains.

Expression and protein structure–activity analysis in the plant kingdom indicated that SBE plays a critical role in affecting the fine structure of amylopectin (Satoh et al., 2003). In another study of developing lotus seeds, a similar conclusion was drawn. In this study, we learned that NnSBEI belong to the SBE B family and tend to transfer longer chains, while NnSBEIII belonged to the SBE A family and prioritized short chain. Higher catalytic activity and expression level of NnSBEI indicated that the long chain was preferred in the transfer during the starch synthesis of lotus seed. Zheng, Zheng & Zeng’s (2004) research about the molecular structure of lotus seed found that the branching degree of amylopectin was short, and the glucose residues of side chains were longer, more than 30. This is consistent with the level of genetic research on *NnSBE*s. Therefore, transcription and activity of SBEs in lotus have a great influence in the fine structure of amylopectin. Furthermore, the edible quality of lotus seed was affected, making it easy for starch to retrogradate.

## Conclusion

This study undertook the preliminary study of *NnSBE* genes in lotus. Two isoforms which encoding starch branching enzyme, were isolated and characterized from lotus. Genetic diversity was analyzed by SNPs of two *NnSBE*s, and revealed the genetic variation levels among varieties. Difference of expression patterns and the affinity about two isozymes were described, as well as the enzyme activity of SBE in development and different tissues, provided necessary information for understanding of the processes involved in starch synthesis from the level of gene and protein. This study revealed the selection of *NnSBE* genes during the cultivation process of lotus, and the effect of *NnSBE* genes on the fine structure of starch in lotus seed. Although the relationships between transcription level, enzyme activity and starch accumulation are complex, our study provides us as much functional information as possible.

##  Supplemental Information

10.7717/peerj.7750/supp-1 Supplemental Information 1 The names and SNPs of 45 individual lotus of four clusters

## Additional Information and Declarations

### Data Availability

The following information was supplied regarding data availability: The SNP data is available in the Supplemental File.
